# Tolerance induction through early feeding to prevent food allergy in infants and children with sensitization against food allergens (TIFFANI): rationale, study design, and methods of a randomized controlled trial

**DOI:** 10.1186/s13063-024-08114-9

**Published:** 2024-04-19

**Authors:** Birgit Kalb, Lara Meixner, Stephanie Heller, Sabine Dölle-Bierke, Stephanie Roll, Tatjana Tissen-Diabaté, Susanne Lau, Sofia Forslund, Ingo Marenholz, Young-Ae Lee, Andreas Thiel, Magda Babina, Jörg Scheffel, Margitta Worm, Kirsten Beyer

**Affiliations:** 1 Department of Pediatric Respiratory Medicine, Immunology and Critical Care Medicine, Charité – Universitätsmedizin Berlin, corporate member of Freie Universität Berlin and Humboldt-Universität Zu Berlin, Berlin, Germany; 2 Division of Allergy and Immunology, Department of Dermatology, Venerology and Allergy, Charité – Universitätsmedizin Berlin, corporate member of Freie Universität Berlin and Humboldt-Universität Zu Berlin, Berlin, Germany; 3 Institute of Social Medicine, Epidemiology and Health Economics, , Charité – Universitätsmedizin Berlin, corporate member of Freie Universität Berlin and Humboldt-Universität Zu Berlin, Berlin, Germany; 4 Experimental and Clinical Research Center, a cooperation between the Max-Delbrück-Center for Molecular Medicine in the Helmholtz Association and the Charité –, Universitätsmedizin Berlin, Berlin, Germany; 5 DZHK (German Centre for Cardiovascular Research), Berlin, Germany; 6 Structural and Computational Biology Unit, EMBL, Heidelberg, Germany; 7 Si-M/“Der Simulierte Mensch” a science framework of Technische Universität Berlin and Charité – , Universitätsmedizin Berlin, Berlin, Germany; 8 Institute of Allergology, Charité – Universitätsmedizin Berlin, a corporate member of Freie Universität Berlin, Humboldt-Universität Zu Berlin and Berlin Institute of Health, Berlin, Germany; 9 Fraunhofer Institute for Translational Medicine and Pharmacology, Allergology and Immunology, Berlin, Germany; 10 BIH Center for Immunomics, Charité – Universitätsmedizin Berlin, corporate member of Freie Universität Berlin and Humboldt-Universität Zu Berlin, Berlin, Germany

**Keywords:** Food allergy, Sensitization, Eczema, Prevention, Early allergen introduction, Window of opportunity

## Abstract

**Background:**

Children with sensitization against foods have to be orally food-challenged before eating these foods for the first time. However, the waiting time for an oral food challenge (OFC) in Germany is about 3–6 months. In contrast, there are hints that an early introduction of allergenic foods might be protective regarding the development of food allergy. The aim of this clinical trial is therefore to investigate, whether an introduction and regular consumption of small amounts of food allergens is safe and will result in an increase of tolerance in children with sensitization against food allergens with unknown clinical relevance.

**Methods:**

In this randomized, placebo-controlled, double-blind, single-center trial, 138 children (8 months to 4 years of age) sensitized to the target allergen(s) hen’s egg, cow’s milk, peanuts, and/or hazelnuts with unknown clinical relevance will be randomized in a 1:1 ratio to either an active or a placebo group, daily receiving a rusk-like biscuit powder with or without the target allergen(s) for 3–6 months until an OFC will be performed in routine diagnostics. The primary endpoint is an IgE-mediated food allergy to the primary target allergen, after the interventional period.

**Discussion:**

Children with sensitization against food allergens with unknown clinical relevance often have to avoid the corresponding foods for several months until an OFC is performed. Therefore, the “window of opportunity” for an early preventive introduction of allergenic foods might be missed. This trial will assess whether an introduction of small allergen amounts will favor tolerance development in these children.

**Trial registration:**

German Clinical Trials Register DRKS00032769. Registered on 02 October 2023.

## Introduction

### Background and rationale {6a}

Up to 8% of all infants and children in industrialized countries suffer from food allergy with cow’s milk, hen’s egg, peanuts, and tree nuts being the main elicitors [1]. Infants with eczema are at particularly high risk for developing food allergies [2]. About half of children with eczema become sensitized, and about one-third will develop food allergy [1, 3, 4]. Many children show allergic reactions upon eating allergenic foods for the first time. The current understanding is that sensitization occurs via the cutaneous route due to an impaired skin barrier in these infants [2]. In the past, prevention strategies for food allergy have been driven by avoidance of allergenic foods in high-risk infants. Despite these attempts, a rising prevalence of food allergy has been observed [1, 5]. On the other hand, it has been shown that an early oral introduction of peanuts and baked hen’s egg can prevent the development of food allergies [6, 7]. Therefore, the current international as well as German guidelines, updated in 2021 and 2022, no longer recommend avoidance of highly allergenic foods [8, 9]. In contrast, for the prevention of hen’s egg allergy, well-cooked egg (e.g., baked or hard-boiled) should be early introduced with the complementary food and given regularly [8, 9]. However, so far, only a protective effect against allergy to well-cooked but not all kinds of egg preparations was shown [6]. For the prevention of peanut allergy, the international guidelines recommend an early introduction of peanuts for infants in countries with a high prevalence of peanut allergy [8]. In Germany, the introduction and regular consumption of peanuts in an age-appropriate form (e.g., peanut butter) may be considered in infants with atopic dermatitis living in families with regular peanut consumption [9]. Beforehand, peanut allergy should be ruled out, especially in infants with moderate to severe atopic dermatitis [9]. Therefore, the infant needs to be tested for sensitization (determination of specific IgE and/or skin prick test), and in case of sensitization, an oral food challenge (OFC) is recommended in order to determine its clinical relevance [10]. This crucial step in the diagnostic workup usually takes several months due to limited capacity for OFCs, because it is a time- and cost-consuming procedure, which can only be performed in specialized centers. Currently, the waiting time for an OFC throughout Germany is about 3–6 months. This is of high concern as the “window of opportunity” for the introduction of food allergens like peanuts and hen’s egg might be missed.

Moreover, for the prevention of other common food allergies in early childhood such as cow’s milk or hazelnut allergy, the best way of allergen introduction into the infant’s diet is still under debate [8, 9].

Therefore, the aim of this trial is to assess if the introduction and regular consumption of small amounts of cow’s milk, hen’s egg, peanuts, and/or hazelnuts is safe and effective in increasing tolerance in children of 8 months to 4 years of age with sensitization against these food allergens, which they have not consumed so far (sensitization without known clinical relevance).

This is an abridged protocol based on protocol version 1.0 dated 29 September 2023. The full protocol adheres to the Standard Protocol Items: Recommendations for Interventional Trials (SPIRIT) recommendations for interventional trials [11].

### Objectives {7}

The primary trial objective is to investigate the impact of an early introduction and continuous intake of small amounts of the target allergenic foods (cow’s milk, hen’s egg, peanuts, and/or hazelnuts) on the development of food allergy in sensitized children with unknown clinical relevance. Secondary objectives are to investigate if the introduction and continuous intake of small amounts of the target allergenic foods is safe in children with food sensitization of unknown clinical relevance, or if it results in an increased occurrence of allergic reactions or gastrointestinal problems. Moreover, the impact of an introduction and continuous intake of small amounts of the target allergenic foods on the development of multiple food allergies, the severity of eczema, the prevalence of asthma, and the immunoglobulin pattern in children with sensitization without known clinical relevance will be assessed.

### Trial design {8}

**Fig. 1:**
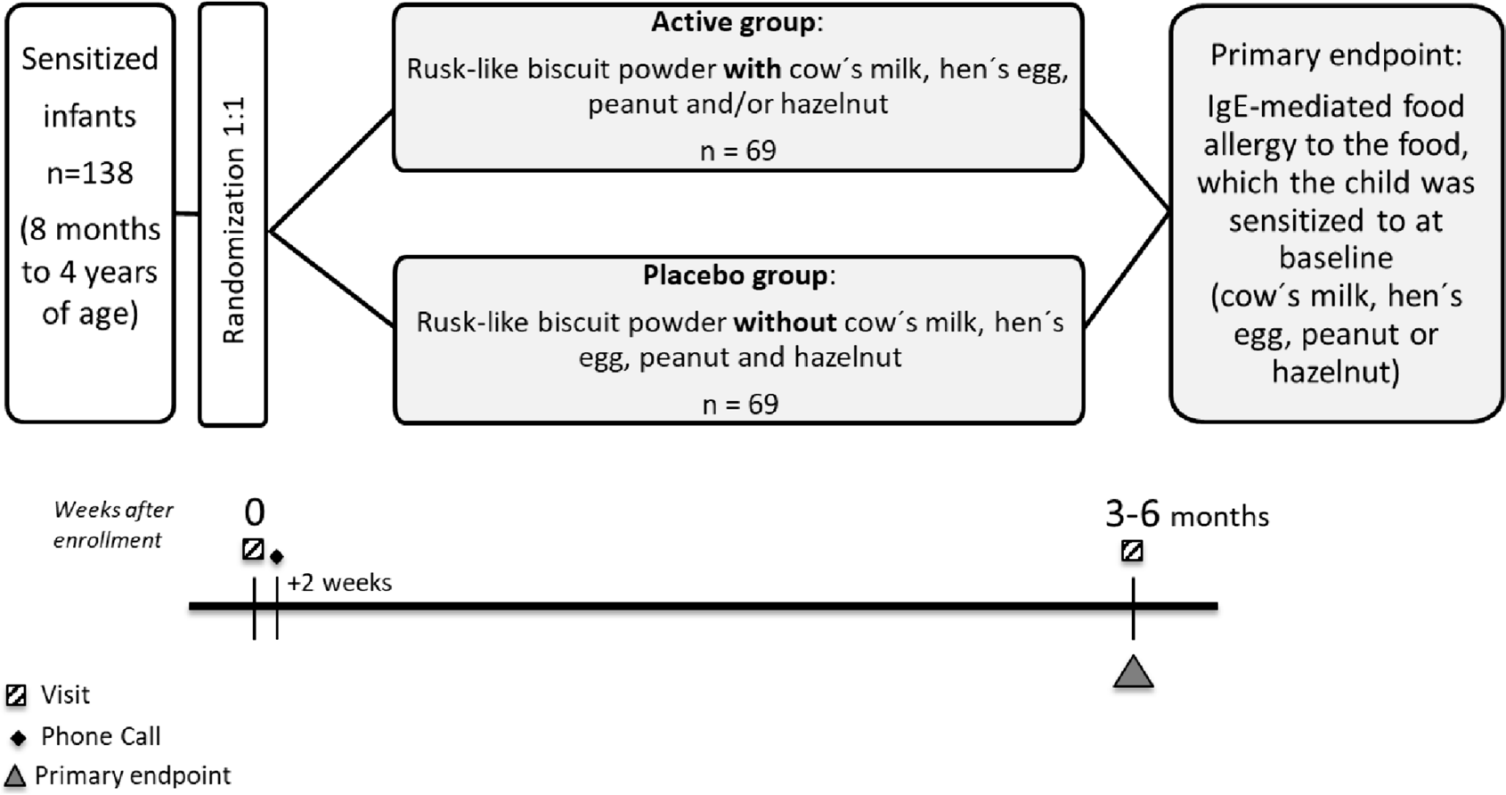
Study design overview. The diagram shows the flow of the patients after enrollment and randomization in the active group or the placebo group. Subjects in the active group will receive a sugar-free rusk-like biscuit powder(s) containing the target food(s) (hen’s egg, cow’s milk, peanuts, and/or hazelnuts) against which they are sensitized with unknown clinical relevance while subjects in the placebo group will receive a sugar-free rusk-like biscuit powder(s) without any of these allergens. After 3 to 6 months of intervention, all subjects will undergo an oral food challenge against the target food(s)

This is a randomized, placebo-controlled, double-blind, superiority, single-center trial with two parallel groups (Fig. 1). Allocation of subjects will be performed in a concealed fashion by authorized site personnel using a REDCap database. Each participant will be allocated to 1 out of 20 kit codes for the corresponding allergen(s) for the interventional product (10 codes active, 10 codes placebo).

TIFFANI is part of the clinical research unit (CRU) 339 “food allergy and tolerance” (FOOD@).

## Methods: participants, interventions, and outcomes

### Study setting {9}

This single-center trial is recruiting participants at the inpatient and outpatient unit of the Department of Pediatric Respiratory Medicine, Immunology and Critical Care Medicine Charité – Universitätsmedizin Berlin. All visits throughout the study will take place either in the study center (visit 1) or on the ward (visit 2: oral food challenge).

### Eligibility criteria {10}

Infants and children aged 8 months to 4 years with a sensitization (specific IgE (sIgE) ≥ 0.1 kU/l and/or skin prick test (SPT) wheal size ≥ 3 mm) against cow’s milk, hen’s egg, peanuts, and/or hazelnuts (target allergenic foods) and an already scheduled oral food challenge at the Charité – Universitätsmedizin Berlin (3–6 months after enrollment) for routine diagnostics are eligible for the trial. Infants are excluded from the study if they have previously consumed the target allergenic food they are sensitized to in relevant amounts. Further exclusion criteria are wheat allergy (study product contains wheat), severe other health issues (e.g., heart disease), a twin sibling already taking part in the study, and participation in another interventional trial.

### Who will take informed consent? {26a}

The legal representative(s) of all participants must read, sign, and date the informed consent form before entering the study or undergoing any study-specific procedures. Before consent is given, the investigator or his/her representative will explain verbally the aim, method, source of funding, and the anticipated benefits and potential risks of the study to the parents; answer all questions regarding the study; and document the informed consent process.

### Additional consent provisions for collection and use of participant data and biological specimens {26b}

As opt-it consent, included in the main study consent form, biological specimens are collected and partially stored for future analysis.

### Interventions

#### Explanation for the choice of comparators {6b}

For eligible children (with sensitization against food allergens with unknown clinical relevance), an OFC is recommended in routine care. Until the OFC is conducted, the corresponding foods have to be avoided. This procedure is the current standard of care and corresponds to the recommendations in the placebo group of this trial.

#### Intervention description {11a}

In the active group, subjects consume daily 3 g of a sugar-free rusk-like biscuit powder, which contains small amounts of cow’s milk, hen’s egg, peanuts, and/or hazelnuts separately, corresponding to their sensitization(s). Therefore, a child may receive only one or up to four foods, each offered in a separate study powder. The allergen dose in each study product will be around 2 mg of food protein (Table 1).

**Table 1:** Allergen amount in study product

	**Interventional allergens**			
	**Peanut**	**Hazelnut**	**Hen’s egg**	**Cow’s milk**
**Amount of protein [mg/3 g] to be taken once daily**	1.7	1.9	1.8	2.0

The sugar-free, rusk-like biscuit powder (3 g of powder/day using a measuring spoon) will be mixed with water, and optionally fruits, to a puree. The study product should be eaten daily throughout the study duration. The first feeding of the study product at visit 1 (V1) will be performed under medical supervision at the study center. The duration of the intervention is determined by the date of the routinely scheduled OFC at visit 2 (V2) and should be at least 3 months and up to 6 months after inclusion to the trial. In case of a postponed OFC (e.g., due to illness of the child), the duration of the intervention can be extended up to a maximum of 9 months.

In the placebo group, subjects consume daily the same sugar-free rusk-like biscuit powder, however, without cow’s milk, hen’s egg, peanuts, or hazelnuts following the same instructions as the active group. To ensure blinding, the first feeding of the placebo study product will also be performed under medical supervision at the study site.

In infants, first, weaning foods (e.g., vegetables) should have been successfully introduced for at least 1 week before feeding the study product. Mothers will be encouraged to continue breastfeeding while introducing weaning foods. Parents of both groups will be advised to avoid cow’s milk, hen’s egg, peanuts, hazelnuts, or their products in their child’s diet according to the individual sensitization profile.

#### Criteria for discontinuing or modifying allocated interventions {11b}

If the subject is unable to consume the recommended amount of rusk-like biscuit powder, e.g., due to feeding difficulties when weaning foods are introduced, the investigator and study physician may adjust the amount and/or dosing regimen. The investigators will prematurely discontinue the study intervention for a subject in case of significant health risk, i.e., after severe adverse events related to the study intervention requiring intensive care treatment, or non-compliance. In case of an intervention with more than one allergen, the intervention will be performed with separate study products. Therefore, in case of study product-related adverse events, intervention may only be discontinued of the corresponding product, whereas the other product(s) could still be offered.

#### Strategies to improve adherence to interventions {11c}

The parents of the study participants will be asked to maintain a weekly (e-)diary from V1 onwards to document consumption and any adverse events. In case of e-diaries, data are stored directly in a central database REDCap. Furthermore, patients will be called once during the study (phone call (PC)) to investigate compliance and tolerability of the regimen. They will be instructed to contact the investigation site by phone in the case of any objective immediate-type allergic reaction occurring within 2 h after food consumption including accidental allergic reactions to other food allergens. Also at V2, compliance, tolerability, and the entries of the (e-)diary will be reviewed and discussed with the investigator or delegated study staff.

#### Relevant concomitant care permitted or prohibited during the trial {11d}

All participants may continue their usual medications, as well as those taken for any concomitant diseases including wheezing and eczema throughout the study. All subjects who will undergo an oral food challenge at V2 will be advised to discontinue oral antihistamines 3–5 days before this procedure.

#### Provisions for post-trial care {30}

Participants who developed a clinically relevant food allergy after completion of the trial will receive individualized dietary counseling. Moreover, parents are offered to contact the outpatient clinic of the Department of Pediatric Pulmonology, Immunology, and Critical Care Medicine, Charité – Universitätsmedizin Berlin for further routine consultation.

### Outcomes {12}

#### Primary endpoint

The primary endpoint is IgE-mediated food allergy to the target food (hen’s egg, cow’s milk, peanuts, or hazelnuts), after 3–6 months of intervention (plus max. 3 months in case of postponed oral food challenge). For polyvalent-sensitized children, a “primary target food allergen” will be determined randomly at enrollment which will be used to define the primary endpoint.

The presence of IgE-mediated food allergy is defined by either of the following (Table 2):

- Positive oral food challenge (plus sIgE ≥ 0.1 or positive skin prick test)
- An immediate-type allergic reaction due to an accidental exposure (plus sIgE ≥ 0.1 or positive skin prick test)

**Table 2:** Diagnosis of food allergy for the determination of endpoints

Procedure	Positive result
**Oral food challenge (OFC)**	OFC outcome is positive if objective symptoms of immediate type allergic reactions occur after any administered dose. (OFC outcome is negative if no objective symptoms of immediate type allergic reactions occur after ingestion of any administered dose.)
**Allergen-specific IgE (sIgE)**	sIgE ≥ 0.1 kU/l
**Skin prick test (SPT)**	Wheel size ≥ 3 mm
**Immediate-type allergic reaction due to accidental exposure**	Parent-reported objective symptoms of immediate type allergic reactions upon accidental exposure to the interventional food(s) during the interventional period

OFC, sIgE determination (blood sampling), and skin prick test (SPT) are performed onsite on the day of the scheduled visit (V2), which should be 3–6 months (plus max. 3 months in case of postponed oral food challenge) after the start of the intervention (V1). Specific IgE and/or SPT at baseline must be assessed no longer than 3 months prior to enrollment.

#### Secondary endpoints

The following secondary endpoints will be assessed after 3–6 months (plus max. 3 months) of intervention (V2):

1. IgE-mediated food allergy determined by OFC (plus sIgE ≥ 0.1 or positive SPT) to the “primary target food allergen”
2. IgE-mediated food allergy determined by an immediate-type allergic reaction due to accidental exposure (plus sIgE ≥ 0.1 or positive SPT) to the “primary target food allergen”
3. IgE-mediated food allergy determined by OFC (plus sIgE ≥ 0.1 or positive SPT) or an immediate-type allergic reaction due to an accidental exposure (plus sIgE ≥ 0.1 or positive SPT) to either of the following (for children sensitized to the respective allergen at baseline):
  - Hen’s egg
  - Cow’s milk
  - Peanuts
  - Hazelnuts
4. Occurrence (frequency and severity) of immediate type allergic symptoms
5. Occurrence of gastrointestinal symptoms
6. Multiple food allergies
7. Number of food allergies
8. Change to baseline of SCORAD (in subjects with eczema)
9. Change to baseline of EASIscore (in subjects with eczema)
10. Wheezing
11. Allergen-specific IgE to hen’s egg, cow’s milk, peanuts, and/or hazelnuts
12. Wheal size measured by skin prick testing to hen’s egg, cow’s milk, peanuts, and/or hazelnuts

#### Participant timeline {13}

During the screening visit (V1), the participation criteria will be checked, and information on demographics, subject/family characteristics, relevant medical history, medication, and nutrition will be recorded. In addition, anthropometric measurements and a physical examination will be performed including the assessment of the severity of eczema (in case of eczema). Skin swabs and stool and saliva samples will be collected. Blood will be collected, or a skin prick test (SPT) will be performed, if the child’s sensitization status was assessed ≥ 3 months prior to V1. Transepidermal water loss (TEWL) will be measured, and palmar hyperlinearity will be determined. Parents are asked to collect a dust sample from the bed and living room within 24 h prior to both study visits. Eligible children will be randomized to one of the two study groups. The first feeding of the study product will be performed at the study site. The study product will be dispensed. Parents will be asked to fill out a (e-)diary for the entire study period (from V1 to V2). Two weeks after V1, parents will be called (PC1) to review compliance, tolerance of the study product, and adverse events. The study product will be consumed daily until an oral food challenge in routine diagnostics at V2 will be performed after their individual waiting time of 3–6 months. At V2, information on medical history, medication, and nutrition will be recorded. In addition, anthropometric measurements and a physical examination will be performed. Skin swabs and stool and saliva samples will be collected. TEWL will be measured. A blood sample will be collected, and a SPT with cow’s milk, hen’s egg, peanuts, and hazelnuts will be performed. Oral food challenges will be performed according to standardized procedures in a double-blinded placebo-controlled manner (Table 3).

**Table 3:** Study visits and procedures

	**Visit 1 (V1)**	**Phone call 1 (PC1)**	**Visit 2 (V2)**
Time	0 weeks, baseline	2 weeks (± 5 days) after V1	3–6 months after V1 ± 14 days
Informed consent	x		
Check eligibility	x		
Randomization	x		
Demographics/subject characteristics	x		x
Family characteristics	x		x
Medical history	x		x
Review adverse events (AEs)		x	x
Review concomitant medication	x	x	x
Nutritional characteristics	x		x
Physical examination	x		x
SCORAD, EASIscore (in case of eczema)	x		x
Transepidermal water loss measurement (TEWL)	x		x
Palmar hyperlinearity	X		
Skin prick testing	(x)^a^		x
Skin swabs	x		x
Saliva sampling	x		x
Stool sampling	x		x
Dust sampling	x		x
Blood sampling	(x)^a^		x
Oral food challenge			x
Dispensation of the study product	x		
Feeding of the study product at the site	x		
Review tolerance study product		x	x

^a^Blood will be collected, or a skin prick test will be performed at V1, if the child’s sensitization status was assessed ≥ 3 months prior to enrollment

#### Sample size {14}

A total of 138 children are planned to be included in the trial. Analyzing 124 children (62 in each group) will result in at least 80% power assuming a difference in the primary endpoint (food allergy after 3–6 months (plus max. 3 months) of 35% in the active group vs. 60% in the control group, based on a two-sided chi-squared test with significance level 5% (two-sided). To compensate for a potential dropout rate of about 10%, 138 infants will be randomized (69 children in each group). Sample size calculation was performed in SAS for Windows version 9.4 (SAS Institute, Cary, NC, USA).

#### Recruitment {15}

Recruitment of participants will take place at the inpatient and outpatient unit of the Department of Pediatric Respiratory Medicine, Immunology, and Critical Care Medicine of the Charité – Universitätsmedizin Berlin. All parents of children with a scheduled OFC with cow’s milk, hen’s egg, peanuts, and/or hazelnuts who have not previously consumed the food they are sensitized to in relevant amounts will be considered for participation. Additionally, we aim to approach participants via flyer advertisement, advertisement in journals, magazines, and social media as well as at information events for (prospective) parents.

### Assignment of interventions: allocation

#### Sequence generation {16a}

After enrollment performed by the study investigators, participants will be randomly assigned to receive verum or placebo. The randomization will be prepared by the trial statistician and will be implemented within the REDCap database system by the data manager. Children will be randomized to receive verum or placebo (1:1 allocation ratio) for each food allergen against which the child is sensitized.

Randomization will be performed in blocks (with varying block lengths) and will be stratified by the following:

- Primary allergen (cow’s milk, hen’s egg, peanuts, or hazelnuts)
- Age (≤ 2 vs. > 2 years of age)
- Grade of sensitization to the primary food allergen (low vs. medium vs. high) defined by either of the following:
  - ◦ Specific IgE levels of the primary food allergen, according to the following CAP classes:
    - Low: IgE ≥ 0.10 ≤ 3.50 (CAP 0–2)
    - Medium: IgE > 3.50 ≤ 50.0 (CAP 3 and 4)
    - High: IgE > 50.0 (CAP 5 and 6)
  - ◦ According to skin prick test results of the primary food allergen (in case of missing specific IgE values)
    - Low: wheal size ≥ 3 mm ≤ 5 mm
    - Medium: wheal size > 5 mm ≤ 9 mm
    - High: wheal size > 9 mm
- Planned duration of intervention, determined by the date of the routinely scheduled OFC (< 5 vs. ≥ 5 months)

#### Concealment mechanism {16b}

Allocation of subjects will be performed concealed (i.e., without knowledge of group allocation) using REDCap where each participant will be allocated to 1 out of 20 kit codes for the corresponding allergen(s) for the interventional product (10 codes active, 10 codes placebo).

#### Implementation {16c}

After enrollment performed by the study investigators, participants will be randomly assigned to receive verum or placebo. The study product will be labeled with the kit codes by staff, who is unblinded, but not otherwise involved in the study.

### Assignment of interventions: blinding

#### Who will be blinded {17a}

Study investigators, all involved study staff, participants, and their parents are blinded to whether the participants will receive allergen-containing rusk-like biscuit powder or allergen-free rusk-like biscuit powder (placebo powder). The main (confirmatory) statistical analysis will be performed blinded to treatment allocation. Unblinding per subject will be performed at the end of the study.

#### Procedure for unblinding if needed {17b}

In case of serious allergic reactions suspected to be related to the study intervention, unblinding will be performed at the discretion of the investigator. To ensure the unblinding process, emergency envelopes, providing information on the individual interventional group the subject is assigned to, are stored at the study center. Once unblinding has been performed, it will be documented in the source data, reported to the PI, and will result in discontinuation of intervention.

### Data collection and management

#### Plans for assessment and collection of outcomes {18a}

Assessment and collection of data and biosamples will be performed by the trained study staff according to standard operating procedures (SOPs). During V1, the participation criteria will be checked and information on demographics, subject/family characteristics, relevant medical history, medication, and nutrition will be recorded. In addition, anthropometric measurements and a physical examination will be performed including the assessment of the severity of eczema by SCORAD [12] and EASIscore [13] in case of eczema. Skin swabs and saliva samples will be collected. Skin swabs are collected and preserved in DNA/RNA shield collection tubes containing medium (DNA/RNA Shield; Zymo Research, Irvine, CA, USA). Saliva samples are collected both with Zymo Swabs as well as with Salimetric Swabs (SalivaBio Swab; Suffolk, Great Britain). The investigator will instruct the parents to collect stool samples (using OMNIgene-GUT tubes, OMR-200; DNA Genotek, Ontario, Canada) from the infants at home. Blood will be collected, or a SPT (all extracts including positive and negative controls: ALK Abelló, Germany) will be performed, if the child’s sensitization status was assessed ≥ 3 months prior to V1. Transepidermal water loss (TEWL) measurement will be performed with the Tewameter TM Hex (Courage + Khazaka Electronic GmbH, Germany). Palmar linearity pattern will be analyzed and documented by palm photographs. The parents should further collect a dust sample using dustream® collector DU-ST-1 (Indoor Biotechnologies LTD, Cardiff, UK) from the child’s bed (or the parent’s bed, if the child sleeps in this bed four nights or more) and the living room. At V2, information on medical history, medication, and nutrition will be recorded. In addition, anthropometric measurements and a physical examination including SCORAD [12] and EASIscore [13] in case of eczema will be performed. Skin swabs and stool and saliva samples will be collected. TEWL will be measured. Moreover, a blood sample will be collected, and a SPT with hen’s egg, cow’s milk, peanuts, and hazelnuts will be performed. OFC will be performed in a double-blinded, placebo-controlled manner in accordance with the clinical routine practice of the Charité – Universitätsmedizin Berlin, based on the PRACTALL international guidelines for OFC and stopped using standardized stopping criteria based on PRACTALL guidelines [14]. Roasted, defatted peanut flour; defatted hazelnut flour; fresh cow’s milk; and pasteurized raw hen’s egg will be used for OFC. Up to seven increasing dose steps at 30-min intervals using a semi-log scale ranging from approximately 2 mg to 3 g food protein (depending on the individual allergen) will be administered. In case of absence of any objective, allergic symptoms, a cumulative dose of 4.4 g of food protein will be administered on another day. To patients with allergic reactions to raw hen’s egg, an additional food challenge with baked hen’s egg will be offered.

#### Plans to promote participant retention and complete follow-up {18b}

Two weeks after V1, parents will be called (PC1) to investigate compliance and tolerability of the study product. In case of discontinuation of the intervention or deviation from the study protocol, participants are encouraged to maintain the scheduled visits or phone calls, and collection of all possible data is planned.

#### Data management {19}

Research Electronic Data Capture (REDCap) will be used as an electronic case report form (eCRF) to collect and manage the study data. REDCap is hosted at the Charité – Universitätsmedizin Berlin and provides an interface for data entry for clinicians. An audit trail will be integrated for tracking data entries and corrections. Data access and storage will follow the data security concept of the Charité including firewalls on the campus level, institutional level, and individual computer level and password-protected access to all computers and folders, which contain sensitive data.

#### Confidentiality {27}

Participant’s privacy and confidentiality will be respected throughout the study. To ensure the protection of personal data, the national legal requirements including the EU General Data Protection Regulation (GDPR) regarding data confidentiality will be followed. Appropriate consent for collection, use, disclosure, and/or transfer (if applicable) of personal information must be obtained in accordance with local data protection laws. A unique participant identifier will be allocated to each participant and assigned chronologically prior to proceeding with study screening. These participant identifiers rather than names will be used to collect, store, and report participant information, including documentation in the eCRF. If the participant’s name appears on any other document (e.g., laboratory report), it must be obliterated on the copy of the document to be, e.g., uploaded to the eCRF. The investigator must retain records and documents, including signed informed consent forms, pertaining to the conduct of this study for 15 years after study completion and final publication. No records may be destroyed during the retention period without the written approval of the sponsor.

#### Plans for collection, laboratory evaluation, and storage of biological specimens for genetic or molecular analysis in this trial/future use {33}

Biosamples obtained in this clinical trial will enable the mechanistic sub-projects of the CRU to investigate the mechanism of food allergy and tolerance development within the clinical research unit. In particular, the role of the gastrointestinal as well as skin microbiome, the IgEome, genetic and epigenetic mechanisms, antigen-specific immunologic reactivities as well as inflammatory circuits, and serological biomarkers will be investigated. The methods of these sub-projects will be described elsewhere. Only the participant identifier will be used to collect and store biosamples. Biological specimens like house dust samples or saliva may be partially stored at the Charité – Universitätsmedizin Berlin.

## Statistical methods

### Statistical methods for primary and secondary outcomes {20a}

The primary analysis of the primary endpoint will be performed by logistic regression with (fixed) factor treatment group (verum, placebo regarding the primary allergen) and the stratification factors primary allergen (hen’s egg, cow’s milk, peanuts, hazelnuts), age group (≤ 2, > 2 years of age), grade of sensitization (low, medium, and high), and planned duration of intervention (< 5, ≥ 5 months). In case of stratification groups with very few cases, stratification groups may be combined (e.g., low vs. medium/high grade of sensitization) or the factor omitted from the analysis model; the decision will be finalized, blinded, and described in the statistical analysis plan (SAP). From this model, the odds ratio for verum vs. placebo treatment will be calculated with a 95% confidence interval and a *p*-value for the treatment group comparison. The analysis will be performed on the full analysis set (FAS) based on the intention-to-treat principle without imputation of missing values. The significance level will be set to 0.05 (two-sided). All other analyses will be considered explorative. Several explorative sensitivity analyses will be performed for the primary endpoint: a re-run of the primary analysis model with the per-protocol population (instead of the FAS). In case of relevant differences between the treatment groups with respect to baseline variables, the primary analysis will be repeated with further adjustment variables (FAS population). To account for the potentially varying lengths of follow-up, a Cox proportional hazards regression as well as Poisson regression will be used to analyze the primary endpoint. Explorative analysis of secondary endpoints will follow the same principle as the primary analysis of the primary endpoint, i.e., models with the (fixed) factor treatment group and the stratification factors. Binary endpoints will be analyzed by logistic regression; continuous endpoints will be analyzed by analysis of covariance (ANCOVA) with the respective baseline value (if available) as an additional covariate. Secondary endpoints will be analyzed with the FAS. Regarding safety endpoints, the nature, frequency, and severity of adverse events and safety variables, including serious adverse events, will be summarized descriptively by treatment group (based on the safety population of all participants who received at least one dose of study treatment and have at least one post-baseline safety assessment, analyzed according to the treatment received). Further details will be described in the SAP, which will be finalized prior to data analysis.

### Interim analyses {21b}

No interim analyses for efficacy are planned.

### Methods for additional analyses (e.g., subgroup analyses) {20b}

Several exploratory subgroup analysis will be conducted regarding the following subgroups: age groups (≤ 2 vs. > 2 years of age); grade of sensitization of the primary food allergen (low vs. medium vs. high); sIgE levels of the primary food allergen according to CAP classes, which are classified into low, medium, and high categories (low: IgE ≥ 0.10 ≤ 3.50: CAP 0–2 vs. medium: IgE > 3.50 ≤ 50.0: CAP 3–4 vs. high: IgE > 50.0: CAP 5–6); sensitization levels of the primary food allergen according to skin prick test results, classified into low, medium, and high (low: wheal size ≥ 3 mm ≤ 5 mm, medium: wheal size > 5 mm ≤ 9 mm, and high: wheal size > 9 mm); duration of intervention (< 5 vs. ≥ 5 months); primary allergen (hen’s egg, cow’s milk, peanuts, hazelnuts); children with eczema (yes/no); carrier of mutations in the filaggrin gene (yes/no): infants with at least one parent with eczema (yes/no); and infants with at least one parent with atopic diseases (asthma, allergic rhinoconjunctivitis, and/or eczema) (yes/no). Subgroup analyses will be performed by additionally including the subgroup variable and the interaction term (subgroup × treatment group) into the primary analysis model (FAS population).

### Methods in analysis to handle protocol non-adherence and any statistical methods to handle missing data {20c}

Selected secondary endpoints will also be analyzed with the per-protocol (PP) population. The PP is a subset of the FAS defined without participants with repeated insufficient consumption of the study product (verum), i.e., consumption < 3 times/week for 3 weeks (either during 3 consecutive weeks or three repeated events of 1 week); OFC taking place before 3 months (after starting the intervention); or OFC taking place after more than 9 months (after starting the intervention). As a general strategy, missing data will not be imputed in this study.

### Plans to give access to the full protocol, participant-level data, and statistical code {31c}

Data will be available upon reasonable request with restrictions regarding scientific purpose and data protection.

### Oversight and monitoring

#### Composition of the coordinating center and trial steering committee {5d}

Trial Management Committee members are KB, MW, BK, and SDB. The steering committee of the CRU will advise on the performance of the project throughout the duration of the trial. Clinical trial monitoring is carried out by the Clinical Trial Office of the Clinical Study Center of the Charité – Universitätsmedizin Berlin and Berlin Institute of Health (BIH). Monitoring will be carried out according to the monitoring manual and depends on the enrollment rate and the data quality. Moreover, there is an external scientific advisory board (SAB), whose members are experts in the field of food allergy, including a representative from the patient organization DAAB (German Allergy and Asthma Association) in order to address particular aspects from the patient side.

#### Composition of the data monitoring committee, its role, and reporting structure {21a}

As the overall risk to patients is low, a data monitoring committee will not be conducted.

#### Adverse event reporting and harms {22}

Pre-existing diseases will be recorded in the subject’s file and in the eCRF system under medical and/or allergy history. Subject reported or physician confirmed worsening of these conditions, or a change of prescribed medication will be assessed during the study visit/phone call and documented in the subject’s file/source data. However, worsening of pre-existing diseases will not be considered as AEs, except those categorized as AEs of special interest (AEIs).

For this specific protocol, AEIs include immediate-type allergic reaction after food exposure, worsening of eczema (in case of eczema), and defined gastrointestinal (GI) symptoms (abdominal pain, bloating, vomiting, difficulties swallowing, stool irregularities).

An accidental food allergen exposure is any known or suspected exposure to a food to which the subject is allergic or sensitized without known clinical relevance, whether or not it results in an AEI.

A serious adverse event (SAE) or serious adverse reaction is defined as any untoward medical occurrence that leads to death, is life-threatening, requires inpatient hospitalization (longer than 24 h), or prolongs an existing hospitalization or results in persistent or significant disability or incapacity. Excluded from this definition are hospitalizations as treatments of a pre-existing condition planned before inclusion into the study or elective surgeries, planned before inclusion into or during the study. According to the seriousness grading, the investigators will review each AE. If the criteria for a SAE are met, the clinical research site will follow local procedures according to the GCP guidelines. All expected and unexpected serious adverse events occurring after the subject’s parents/caregivers have signed the informed consent and the intervention has been started must be reported on a SAE form to the study principal investigator within 24 h of becoming aware of the event.

Any serious allergic reaction related to the study intervention will lead to a temporary halt of the study in order to re-evaluate the safety conditions. For this purpose, an external safety advisory board is called in to advise the PI and the CO-PI of the study. Based on the recommendations of this committee, a decision is made as to whether the study can be continued or whether changes to the design need to be made. If three participants experience serious allergic reactions related to the study intervention, the study will be stopped.

AE/AEI recording will extend from enrollment until the end of the study at V2.

Parents of the participants should document all AEs and AEIs occurring during the study in the subject’s (e-)diary. Moreover, the subject’s parents will be instructed to document accidental food allergen exposures in detail in the (e-)diary and to contact the study site after any known or suspected food allergen exposure, even if it does not cause symptoms. In case of an immediate type allergic reaction after food exposure (either to the study product or due to exposure to (other) food allergens) or recurrent GI symptoms, parents of the participants are asked to call the study site. During the phone call and the second study visit, the subject’s parents/caregivers will be queried on AEs/AEIs based on the patient’s diary and changes in the subject’s condition.

For this specific protocol, only AEIs (see above) and SAEs are logged as (S)AEs in the eCRF. Information will be recorded regarding the date of onset, date of resolution, seriousness, severity, outcome, treatment required, causality, action taken with the study intervention if applicable, possible augmentation factors, and/or other possible causes. For scoring the severity of immediate-type allergic reactions, the grading system for food-induced anaphylaxis published by Sampson is used [14].

The investigators will assess the causality/relationship between the study intervention and the AE. Causality will be documented as related or not related to the intake of the study intervention. Overall, the occurrence of immediate type allergic reactions within 2–3 h after food allergen exposure (study intervention and/or accidental exposure) will be considered as related to food exposure.

#### Frequency and plans for auditing trial conduct {23}

An audit is not planned by the sponsor due to the nature of the study. The steering committee of the clinical research unit CRU will advise on the performance of the project throughout the complete duration of the trial. Clinical trial monitoring is carried out by the Clinical Trial Office of the Clinical Study Center of the Charité – Universitätsmedizin Berlin and Berlin Institute of Health (BIH) and will be carried out according to the monitoring manual. The frequency depends on the enrollment rate and the data quality.

#### Plans for communicating important protocol amendments to relevant parties (e.g., trial participants, ethical committees) {25}

All parents give their full consent before their children are enrolled in the trial. The written informed consent must be given by both the mother and father or the legal representatives. Any substantial amendments to the protocol and/or to the consent materials must be approved by the Ethics Committee of Charité – Universitätsmedizin Berlin before implementation. The written informed consent form must be revised whenever important new safety information is available, whenever the protocol is amended leading to changes in study procedures relevant to the patient, and/or whenever any new information becomes available that may affect participation in the trial.

#### Dissemination plans {31a}

Besides the study protocol, publications are planned for the results in peer-reviewed journals. In addition, results will be communicated in lay language to participants and health care providers.

## Discussion

This clinical trial, addressing infants and children at 8 months to 4 years of age, who are sensitized against cow’s milk, hen’s egg, peanuts, and/or hazelnuts with unknown clinical relevance, will assess if an introduction and regular consumption of small amounts of the corresponding food allergen(s) is safe and will promote tolerance development. The study population consists of children with an already scheduled OFC, who will receive a rusk-like biscuit powder containing the target allergen(s) or placebo for 3–6 months until the OFC will be performed in routine diagnostics.

Particularly infants with eczema are at high risk for sensitization against the most important food allergens. In case of sensitization, they have to be orally food-challenged, before these allergens are administered for the first time [15, 16]. But as oral food challenges are expensive and time-consuming, capacities in clinics are limited [17]. As a consequence, the waiting time for an OFC can comprise several months, which may lead to a delayed introduction of food allergens into the children’s diet, but avoidance of food allergens in these children may be of high concern, since the “window of opportunity” for an oral introduction to promote tolerance development might be missed. Although the first 12 months of life seem to be the optimal window of opportunity for tolerance induction, many families do not introduce a broad variety of foods to their infants. Therefore, it is worth evaluating if it is possible to support tolerance induction during a broader timeframe beyond the first year of life.

Within the LEAP trial, the effect of an early introduction of peanuts in high-risk infants with severe eczema and/or hen’s egg allergy was investigated. It has been shown that the early introduction and continuous feeding of peanut products reduced the development of peanut allergy by about 80% [7]. The EAT study examined the effects of an early administration of various foods to breast-fed infants from the general population, starting at 3 months of age. In the per-protocol analysis, the prevalence of any food allergy was significantly lower in the group of children that early introduced the allergens compared to the group with the standard introduction (2.4% versus 7.3%) [18]. By analyzing data from the LEAP and the EAT study as well as from the PAS observational cohort, Roberts et al. could show that the preventive effect of an early allergen introduction decreased with increasing age at introduction. The authors further reported that the impact of a delayed introduction was even more profound in infants with increasing severity of eczema [19]. The PETIT study enrolled infants with eczema at 4 to 5 months of age for receiving small amounts of heated egg-containing study product or placebo for 6 months. At 12 months of age, only 8% of the active group versus 38% of the placebo group suffered from hen’s egg allergy [6]. Interestingly, the subgroup analysis revealed that the intervention seemed to be more effective in infants that were already sensitized against hen’s egg at baseline (risk difference 34.4% [17.0–51.7]; *p* = 0·001) compared to the non-sensitized ones (risk difference 16.7% [95% CI − 10.2 to 43.5]; *p* = 0·31). These findings imply that a delayed administration of food allergens, especially in children that are already sensitized, may be of concern regarding the development of food allergies.

However, besides the timing of introduction and the target group (children at risk versus children from the general population), the amount and the preparation of allergens as well as the duration and frequency of intake may play a role regarding food allergy prevention. Concerning hen’s egg, several interventional studies using pasteurized raw hen’s egg with amounts ranging from 350 mg to 2.5 g showed no significant reduction of hen’s egg allergy due to an early introduction, but high rates of adverse reactions, both immediate type symptoms and gastrointestinal problems [20–22]. In contrast, the PETIT study using small amounts (25 mg for 3 months, then 125 mg for 3 months) of baked hen’s egg could show an effect on the prevention of hen’s egg allergy [6]. Within the LEAP trial, relatively high amounts of peanut protein were administered (6 g per week, distributed in three or more meals), but no major safety issues occurred [7]. Nevertheless, it has to be mentioned that a SPT with peanuts was performed prior to randomization, and infants with a wheal size greater than 4 mm were not randomized [7]. Considering that in the TIFFANI trial, also children with high sIgE levels at baseline will be enrolled, very small amounts of food allergens are used, reflecting the allergen-specific reference doses “ED_05_” (approximately 2 mg food protein of hen’s egg, cow’s milk, hazelnuts, and peanuts). The ED_05_ is defined as the eliciting dose at which 5% of the respective allergic population would be predicted to experience an allergic reaction [23].

Regarding the duration of the intervention, most randomized-controlled studies investigating the effect of an early introduction of food allergens on the development of food allergy, have decided on an interventional period of 6 months or longer. Within the SPADE study, however, two OFCs were performed to examine the preventive effect of an early introduction of small amounts of cow’s milk formula in breast-fed infants from the general population after approximately 2 and 5 months of intervention [24]. After 5 months of intervention, 0.8% of the ingestion group versus 6.8% of the placebo group developed cow’s milk allergy. But interestingly, even after only approximately 2 months of intervention there was a tendency towards a preventive effect of an early cow’s milk introduction (0.4% suffered from cow’s milk allergy in the ingestion group, compared to 2.5% in the avoidance group) [24].

Taken together, within this randomized, placebo-controlled trial, we will investigate if the administration of very small amounts of food allergens (hen’s egg, cow’s milk, peanuts, and/or hazelnuts) for 3 to 6 months is safe and has an effect on the prevention of food allergy in infants and children that are sensitized to the corresponding food allergen(s) with unknown clinical relevance. Results of this study will be crucial in terms of food allergy prevention, since they may help to take advantage of the “window of opportunity” for the introduction of allergenic foods, instead of avoidance while waiting for further diagnostic procedures.

## Trial status

Protocol version 1.0 dated 29 September 2023. The first patient is planned to be randomized in November 2023 and the last patient is planned to be enrolled in October 2024.

## Data Availability

All data generated in the CRU will be curated and organized into a set of files, shared on an online, publicly available data repository after peer-reviewed publication (preservation and accessibility of the data) to ensure potential secondary analyses, long-term archiving, and reuse by other researchers (scientific recognition).

## Abbreviations

AE: Adverse event
AEi: Adverse event of special interest
ANCOVA: Analysis of covariance
BIH: Berlin Institute of Health
CRF: Case report form
CRU: Clinical research unit
DBPCFC: Double-blind placebo-controlled food challenge
DFG: German Research Foundation
EASIscore: Eczema Area and Severity Index Score
EAT study: Enquiring About Tolerance study
eCRF: Electronic case report form
FAS: Full analysis set
GDPR: General Data Protection Regulation
GI: Gastrointestinal
HEAP trial: Hen’s Egg Allergy Prevention trial
IgE: Immunoglobulin E
LEAP trial: Learning Early About Peanut trial
OFC: Oral food challenge
PAS cohort: Peanut Allergy Sensitization cohort
PC: Phone call
PP: Per-protocol
PRACTALL: PRACTical issues of ALLergology, Joint United States/European Union Initiative
REDCap: Research Electronic Data Capture
SAE: Serious adverse event
SAP: Statistical analysis pan
SCORAD: SCORing Atopic Dermatitis
sIgE: Specific immunoglobulin E
SPADE trial: Randomized trial of early infant formula introduction to prevent cow’s milk allergy
SPT: Skin prick test
TEWL: Transepidermal water loss
TIFFANI: Tolerance induction through early feeding to prevent food allergy in infants and children with sensitization against food allergens
V: Visit
